# Climate Change Restructures the Suitable Habitat of *Bambusa emeiensis* in Southwestern China: Disproportionate Core-Habitat Loss and Divergent Centroid Shifts

**DOI:** 10.3390/plants15101575

**Published:** 2026-05-21

**Authors:** Miao Liu, Chunju Cai, Guanglu Liu, Xiaopeng Shi, Shuguang Li, Shaohui Fan

**Affiliations:** 1 International Centre for Bamboo and Rattan, Beijing 100102, China; 2 Key Laboratory of National Forestry and Grassland Administration for Bamboo & Rattan Science and Technology, Beijing 100102, China

**Keywords:** *Bambusa emeiensis*, climate change, species distribution model, BIOMOD2, habitat suitability, centroid shift, core habitat

## Abstract

Bamboo is an ecologically and economically important forest resource in China, and understanding how climate change reshapes bamboo habitat suitability is essential for sustainable cultivation, introduction, and germplasm conservation. *Bambusa emeiensis*, an accepted bamboo species native to southern China and widely cultivated in southwestern China, has important management and utilization value, yet its future habitat dynamics and the stability of its highly suitable core habitats remain poorly understood. To address this gap, an ensemble species distribution modeling framework based on BIOMOD2 was used to predict the current and future suitable habitats of *B. emeiensis* under multiple climate scenarios, identify the dominant environmental constraints, and compare shifts between overall suitable habitat and highly suitable core habitat. The ensemble model showed high discrimination capacity under random cross-validation, but its transferability should be interpreted cautiously because occurrence records may be spatially autocorrelated and the projections remain correlative. Annual temperature range, elevation, and precipitation of the warmest quarter emerged as the strongest statistical predictors of distribution. Under the current climate, suitable habitats were concentrated in southwestern China, especially in the transitional zone spanning southern Sichuan, southwestern Chongqing, and northern Guizhou. Across all six future scenarios examined, the total suitable area declined relative to the current climate, with reductions ranging from about 25% under SSP3-7.0–2090s to more than 50% under SSP5-8.5–2050s, and highly suitable core habitat contracted even more strongly (by 41–95% across scenarios). In addition, centroid shifts of overall suitable habitat were not always synchronized with those of highly suitable core habitat, suggesting that climate change may reorganize not only habitat extent, but also the internal spatial arrangement of optimal environments. These findings indicate that the future management of *B. emeiensis* should prioritize the persistence, connectivity, and managed directional relocation of core habitats rather than relying solely on changes in total suitable area.

## 1. Introduction

Bamboo is one of the most important non-timber forest resources in Asia and plays major roles in carbon sequestration, soil and water conservation, rural livelihoods, ecosystem stability, and green bioeconomy development [1,2]. China harbors the richest bamboo resources in the world, with high species diversity, broad geographic distribution, and substantial ecological and industrial importance [2]. Among Chinese bamboos, *Bambusa emeiensis* is an accepted species recorded from southern China. Standard taxonomic sources, including Flora of China and Plants of the World Online, recognize the species and provide the taxonomic basis for its scientific use in ecological and management studies [3,4].

Climate change is reshaping plant distributions worldwide by altering temperature regimes, precipitation seasonality, disturbance patterns, and the climatic suitability of local habitats [5,6,7]. Range shifts, habitat contraction, community disequilibrium, and centroid displacement have all been widely documented under recent and projected warming [8,9]. Bamboo species may be especially sensitive to such changes because their growth and regeneration are strongly constrained by hydrothermal seasonality, topography, and, in some taxa, long reproductive intervals and limited natural migration capacity [2,10]. Most bamboos, including *B. emeiensis*, are clonal sympodial plants that propagate primarily by rhizome and clump division, with very limited natural seed dispersal [2,10]; for such taxa, climatically suitable patches emerging far from the current range are essentially unreachable on management-relevant timescales without human-mediated planting, which has direct implications for how ‘potentially suitable habitat’ is interpreted (see Section 4.4). Recent studies have shown that climate change may significantly alter the distribution of bamboo forests in China, affect rare and endangered bamboo taxa, and modify bamboo resources associated with giant panda habitats and regional conservation priorities [2,11,12,13,14].

Species distribution models have become standard tools for linking species occurrence records with environmental predictors and projecting suitable habitats under climate change [15,16,17]. Among these approaches, ensemble forecasting is often regarded as more robust than reliance on any single algorithm because it integrates methodological uncertainty and can improve prediction stability [18,19,20]. BIOMOD and BIOMOD2 are therefore widely used for climate-change assessments of plant distributions including recent work on bamboos and woody species in China [21,22]. However, many SDM studies still focus mainly on the total suitable area, while paying less attention to the internal structure of suitable habitat, especially the distinction between overall suitable habitat and highly suitable core habitat [20,23]. This distinction matters because a modest expansion of marginally suitable area does not necessarily imply the persistence of climatically optimal habitats that are most relevant for long-term cultivation, productivity, and conservation.

For *Bambusa emeiensis*, previous work has mainly emphasized taxonomy, cultivation, and broad bamboo-resource context, whereas quantitative assessment of its future habitat dynamics, dominant climatic constraints, and structural reorganization under climate change remains limited. The complex topography of southwestern China further suggests that future responses of *B. emeiensis* may be nonlinear and spatially heterogeneous. Therefore, this study used an ensemble SDM framework based on BIOMOD2 to evaluate model performance and identify key environmental drivers, characterize the current suitable habitat pattern, assess future habitat changes under multiple climate scenarios, and compare centroid shifts between overall suitable habitat and highly suitable core habitat. We hypothesized that future climate change would alter not only the total extent of suitable habitat, but also the internal structure of habitat quality, with highly suitable core habitats showing stronger instability than total suitable habitat.

## 2. Results

### 2.1. Model Performance and Dominant Environmental Drivers

**Figure 1:**
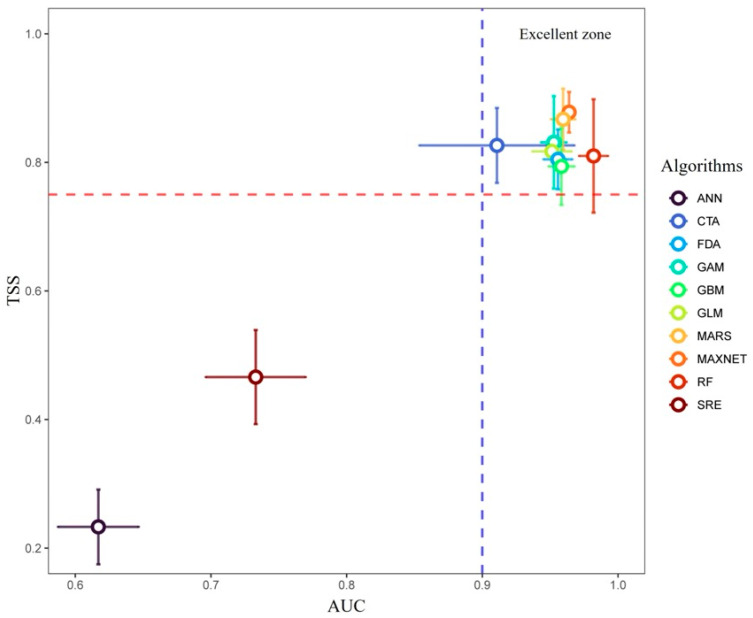
Predictive performance of single algorithms and the weighted-mean ensemble model for *Bambusa emeiensis*. Each colored circle represents the mean performance of one modeling algorithm based on random 75/25 hold-out cross-validation, with three pseudo-absence sets and five repeated data splits per set, yielding 15 evaluation replicates per algorithm. Horizontal and vertical error bars indicate standard deviations of AUC and TSS, respectively. The vertical dashed line marks the commonly used AUC threshold of 0.90 for excellent discrimination, and the horizontal dashed line marks the TSS threshold of 0.75. Algorithms located in the upper-right region therefore showed stronger discrimination capacity and contributed more reliably to the final ensemble model. ANN, CTA, FDA, GAM, GBM, GLM, MARS, MaxEnt, RF, and SRE denote the ten individual algorithms implemented in BIOMOD2.

The individual algorithms differed in predictive performance, but most major models showed strong discrimination capacity, indicating a stable statistical relationship between *Bambusa emeiensis* occurrence records and the selected environmental predictors. Among the single algorithms, MARS, GAM, CTA, GBM, MaxEnt, and RF all performed well under random cross-validation, with high ROC values and generally high TSS values, whereas ANN and SRE performed comparatively poorly. These results justified the use of an ensemble framework for final prediction. The weighted mean ensemble model further improved overall reliability and showed high sensitivity, specificity, and discrimination scores, indicating strong ability to distinguish both suitable and unsuitable areas under the adopted random hold-out evaluation scheme (Table 1 and Table 2; Figure 1). Because random partitioning can be affected by spatial autocorrelation, these values should be interpreted as apparent discrimination performance rather than as direct estimates of spatial transferability under novel future climates (see Section 4.5).

**Figure 2:**
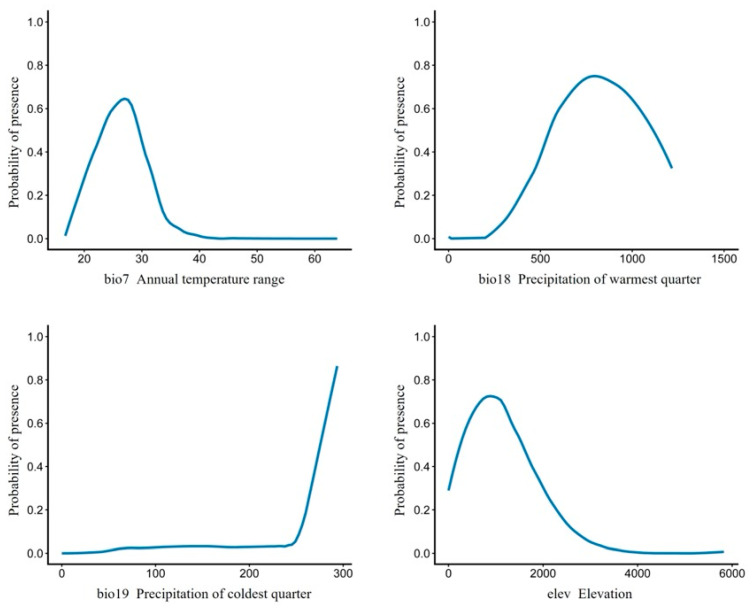
Response curves of *Bambusa emeiensis* to the four retained environmental predictors in the weighted-mean ensemble model. Blue curves show the ensemble-predicted probability of presence when one predictor was varied across its observed range while the other predictors were held at their median values in the calibration data. The four predictors were annual temperature range (bio7), precipitation of the warmest quarter (bio18), precipitation of the coldest quarter (bio19), and elevation (elev). The curves were generated using the BIOMOD2 evaluation-strip procedure after variable screening by Pearson correlation and variance inflation factor analysis. The final modeling dataset contained 187 presence records and three sets of 10,000 pseudo-absences. The sharp increase in the upper tail of bio19 should be interpreted cautiously because this part of the curve is supported by relatively sparse occurrence records and may represent an edge effect rather than a robust ecological threshold.

Variable importance analysis showed that annual temperature range (bio7) was the strongest statistical predictor of *B. emeiensis* distribution in the ensemble, followed by elevation, precipitation of the warmest quarter, and precipitation of the coldest quarter (Table 3). Because annual temperature range covaries with latitude and continentality, this dominance should be interpreted primarily as a statistical association rather than as direct evidence of a specific physiological mechanism; we return to this point in Section 4.1. The response curves revealed a clear ecological preference for moderate annual thermal amplitude, relatively low to mid elevation, and sufficient warm-season precipitation. In contrast, cold-season precipitation (bio19) contributed less strongly and appeared to function more as a secondary constraint than as a primary driver; the abrupt rise visible at the upper tail of the bio19 response curve (above ~250 mm) coincided with sparse sampling at the wettest extreme of the predictor distribution and was regarded as a possible edge artifact rather than as a robust ecological threshold. Together, these results indicate that the realized distribution of *B. emeiensis* is jointly shaped by thermal seasonality, topographic heat availability, and growing-season water supply, rather than by temperature or precipitation alone (Table 3; Figure 2).

### 2.2. Current Habitat Suitability Pattern

**Figure 3:**
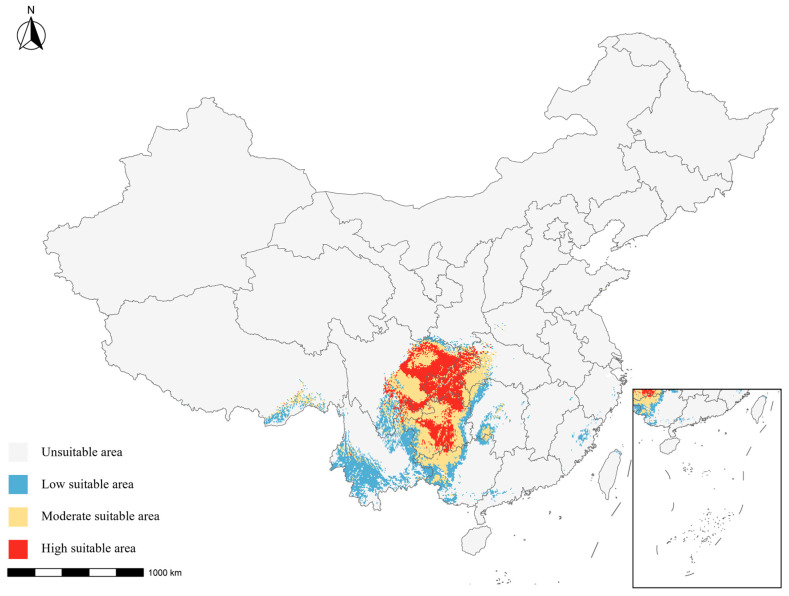
Current potential suitable distribution of *Bambusa emeiensis* under the baseline climate period. Habitat suitability was predicted using the weighted-mean BIOMOD2 ensemble model and classified on the 0–1000 suitability scale. Grey indicates unsuitable area (0–532), blue indicates low suitability (533–650), yellow indicates moderate suitability (651–800), and red indicates high suitability (801–1000). The threshold of 532 was derived from the TSS-maximizing cutoff of the ensemble model, and the same class boundaries were used for all current and future projections to ensure comparability. Area statistics for each suitability class are provided in Table 4. Provincial boundaries and the inset map are shown for geographic reference.

Under the current climate, the suitable habitat of *B. emeiensis* was concentrated in southwestern China and displayed a distinct core-transition-fragmented margin spatial pattern. Highly suitable habitats were mainly located in southern Sichuan, southwestern Chongqing, and adjacent northern Guizhou, forming the principal core of climatic suitability. Moderately suitable habitats were distributed in belts surrounding the core areas and extended toward central Guizhou, northeastern Yunnan, and nearby montane transitional zones. Low-suitability habitats were more fragmented and scattered along peripheral ecotones. Overall, the present pattern was broadly consistent with the known contemporary distribution center of the species (Table 4; Figure 3).

Area statistics under the current climate confirmed that the species occupies a relatively narrow but well-developed climatic niche in southwestern China: unsuitable habitats covered approximately 829.46 × 10^4^ km^2^ (87.37% of the study area), while the total suitable habitat reached 119.89 × 10^4^ km^2^ (12.63%), of which 36.31 × 10^4^ km^2^ (3.82%) corresponded to highly suitable core habitat (Table 4). Although the species exhibited some potential for regional expansion, areas reaching moderate to high suitability remained spatially concentrated rather than widespread. These results support the inference that current suitable habitat is limited, spatially clustered, and centered in southwestern China.

### 2.3. Future Habitat Restructuring Under Multiple Climate Scenarios

**Figure 4:**
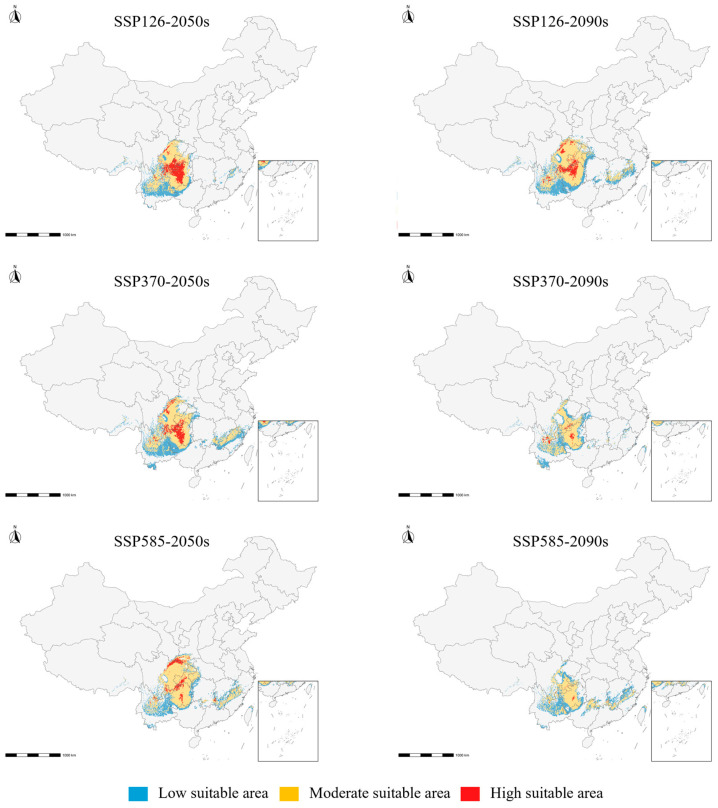
Future potential suitable distribution of *Bambusa emeiensis* under six climate-change scenarios. Maps show the ensemble-projected habitat suitability for three Shared Socioeconomic Pathways, SSP1-2.6, SSP3-7.0, and SSP5-8.5, across two future periods, the 2050s and 2090s. Grey indicates unsuitable area, blue indicates low suitability, yellow indicates moderate suitability, and red indicates high suitability; the same suitability thresholds used in Figure 4 were applied to all scenarios. Future climate layers were based on the multi-GCM mean of BCC-CSM2-MR, MI-ROC6, and CanESM5 at a 2.5-arc-minute resolution, while elevation was kept constant. The maps show that future climate change mainly reduces and fragments highly suitable core habitats, even where some low- or moderate-suitability areas remain. Area changes for each scenario are summarized in Table 5.

Under all six future scenarios examined, the total suitable habitat of *B. emeiensis* declined relative to the current climate, but the magnitude and structure of the decline varied strongly with scenario and time period (Table 5; Figure 4). The total suitable area was reduced by 26.66% under SSP370-2050s and 25.47% under SSP370–2090s, by 29.77% under SSP126–2050s and 40.85% under SSP126–2090s, and most strongly by 52.56% under SSP585–2050s, with a partial rebound to a 27.86% reduction under SSP585–2090s. Future climate change therefore did not produce a simple monotonic trend; rather, it produced a scenario-dependent restructuring superimposed on a general contraction of the suitable range.

More importantly, habitat quality changed even more strongly than the total habitat extent. Across all six future scenarios, the highly suitable core habitat contracted disproportionately, by 41.39% (SSP126–2050s), 67.72% (SSP126–2090s), 78.79% (SSP370–2050s), 75.90% (SSP370–2090s), 95.40% (SSP585–2050s), and 83.78% (SSP585–2090s) relative to the current value of 36.31 × 10^4^ km^2^. In contrast, the moderate-suitability area declined less sharply than high-suitability core habitat and remained close to the current level in some scenarios (e.g., SSP585–2090s, 50.99 × 10^4^ km^2^ vs. 52.23 × 10^4^ km^2^ currently), partially offsetting the loss of high-suitability core in the total-area statistic. This contrast indicates that future climate change is projected to erode the most favorable core habitats currently supporting *B. emeiensis* while leaving a larger fraction of the future suitable area in the moderately suitable category. Future responses of *B. emeiensis* are therefore better interpreted as a restructuring of habitat quality and spatial configuration alongside an overall reduction in suitable area, rather than as simple range expansion or contraction.

The unusually large contraction of highly suitable core habitat under SSP585–2050s (1.67 × 10^4^ km^2^) followed by a partial recovery under SSP585–2090s (5.89 × 10^4^ km^2^) indicates a non-monotonic response of core habitat under the high-emission pathway. Because this pattern was derived from a correlative SDM based on a limited set of GCMs, it should be interpreted as a scenario-specific projection rather than as a deterministic ecological trajectory. The result nevertheless highlights that high-suitability core habitat may be more sensitive and less stable than the total suitable area, especially under strong climate forcing.

Spatially, future suitable habitats remained centered in the mountain-basin transitional zone of southwestern China, but stronger warming and later-period scenarios tended to reduce the connectivity of highly suitable patches and increase fragmentation. This finding implies that even when the future suitable area is not maximally reduced (e.g., SSP370 scenarios), the spatial integrity and management value of the habitat network may still decline. Accordingly, the total habitat area alone is insufficient for evaluating the long-term persistence potential of *B. emeiensis*.

### 2.4. Divergent Centroid Shifts of Overall Suitable Habitat and Highly Suitable Core Habitat

**Figure 5:**
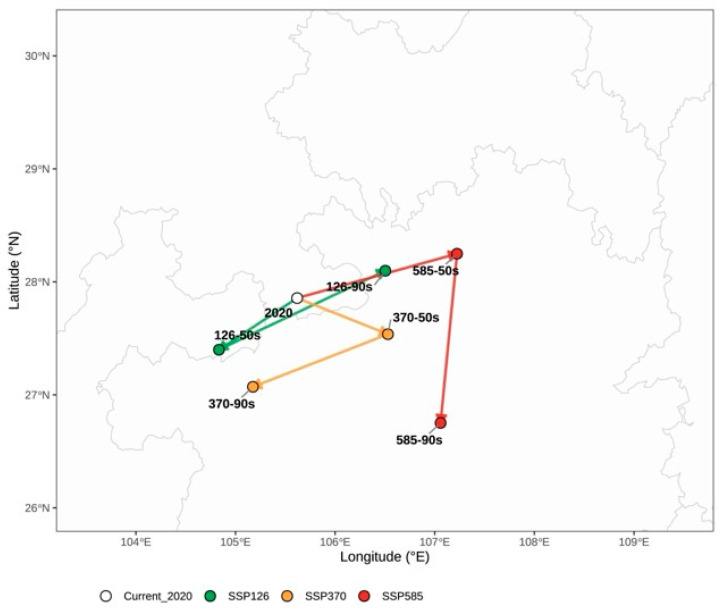
Centroid shifts of the overall suitable habitat of *Bambusa emeiensis* under current and future climate scenarios. The overall suitable habitat includes low-, moderate-, and high-suitability classes combined. The open circle marks the current centroid, green symbols and lines indicate SSP1-2.6 scenarios, yellow/orange symbols and lines indicate SSP3-7.0 scenarios, and red symbols and lines indicate SSP5-8.5 scenarios. Lines connect the current centroid with future centroids for the 2050s and 2090s, showing the direction and magnitude of projected spatial displacement. Centroids were calculated as area-weighted mean centers in an Albers equal-area conic projection and then transformed back to WGS84 coordinates for display. Shift distances and compass directions are reported in Table 6.

Centroid analysis showed that climate change would reorganize not only the outer extent of suitable habitat, but also the spatial arrangement of optimal habitat conditions. The centroid of overall suitable habitat exhibited scenario-dependent directional shifts, with oscillatory movements under different emission pathways and future periods (Table 6; Figure 5). Under stronger warming, the magnitude of overall centroid displacement generally increased, indicating enhanced spatial reorganization of the broader suitable range.

**Figure 6:**
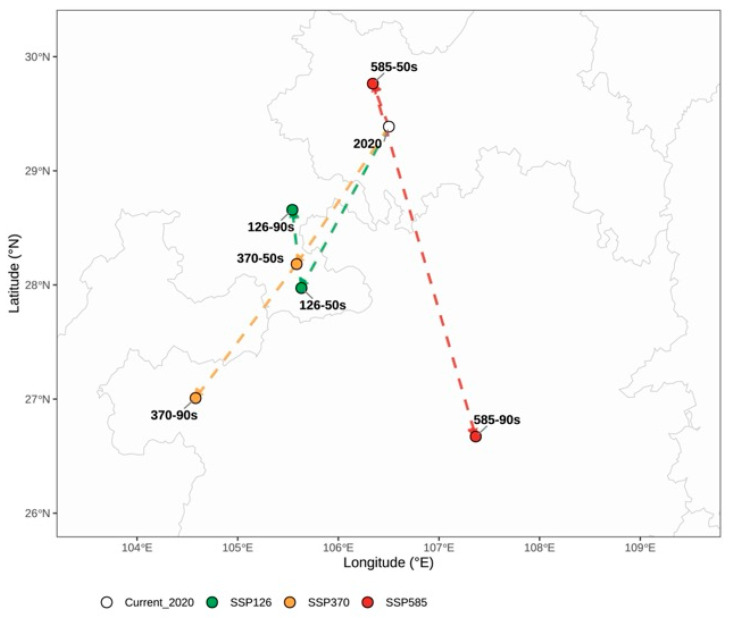
Centroid shifts of the highly suitable core habitat of *Bambusa emeiensis* under current and future climate scenarios. The highly suitable core habitat corresponds only to the high-suitability class, defined as suitability values of 801–1000 on the BIOMOD2 scale. The open circle marks the current core-habitat centroid, green symbols and lines indicate SSP1-2.6 scenarios, yellow/orange symbols and lines indicate SSP3-7.0 scenarios, and red symbols and lines indicate SSP5-8.5 scenarios. Lines connect the current centroid with future core-habitat centroids and indicate projected displacement direction and distance. Compared with Figure 6, this figure focuses on the most climatically favorable habitat fraction rather than the entire suitable range. The SSP5-8.5–2050s centroid should be interpreted cautiously because the highly suitable core area under this scenario was very small, only 1.67 × 10^4^ km^2^, making its centroid sensitive to the spatial configuration of a limited number of highly suitable cells.

The centroid of highly suitable core habitat showed an even more informative pattern. In several scenarios, the core-habitat centroid moved farther than the centroid of the total suitable habitat, and the two were not always synchronized in direction. This mismatch indicates that future climate change may alter not only the amount of suitable habitat but also the internal geography of the most climatically favorable environments. In practical terms, areas that remain suitable in a broad sense may no longer correspond to the most stable or optimal habitats for the long-term cultivation and germplasm conservation of *B. emeiensis* (Figure 6).

Centroid estimates should be interpreted with caution when the underlying highly suitable area becomes very small. In particular, the SSP585–2050s core habitat declined to only 1.67 × 10^4^ km^2^, so the reported 44.64 km northward shift is sensitive to the spatial configuration of a small set of highly suitable cells. This directional label should therefore be regarded as indicative rather than as a precise migration pathway. The broader conclusion is not that *B. emeiensis* will naturally migrate along a single centroid trajectory, but that climate change may reorganize the internal geography of optimal habitat conditions.

## 3. Materials and Methods

### 3.1. Study Species and Occurrence Records

*Bambusa emeiensis* L.C.Chia & H.L.Fung is a clumping (sympodial) bamboo species native to southern China and is widely cultivated in southwestern China. Occurrence records were compiled from three complementary sources: (i) the Global Biodiversity Information Facility (GBIF; accessed on 15 January 2024 via https://www.gbif.org); (ii) the Chinese Virtual Herbarium (CVH; https://www.cvh.ac.cn) and the National Specimen Information Infrastructure (NSII; http://www.nsii.org.cn); and (iii) georeferenced field-survey records contributed by the authors during bamboo-resource investigations in Sichuan, Chongqing, Guizhou, and Yunnan Provinces. Taxonomic validity of the species was confirmed against Flora of China and Plants of the World Online, and synonyms were merged under the accepted name.

Raw records were then subjected to a standardized cleaning pipeline in R 4.3.2 using the CoordinateCleaner 2.0-20 package. Records were removed if they (i) lacked coordinates or had coordinate precision coarser than 0.01° (≈1 km); (ii) fell in the ocean, in country or province centroids, or at known institution or herbarium localities; (iii) had equal longitude and latitude values or zero coordinates; or (iv) were geographically inconsistent with the known distribution of the species (i.e., outside mainland China or outside the elevational range 200–2200 m). Records with collection dates earlier than 1970 were also excluded to match the temporal coverage of the baseline climate dataset. To reduce spatial sampling bias and pseudo-replication, the remaining points were spatially thinned to a minimum nearest-neighbor distance of one raster cell (approximately 4.6 km at the 2.5-arc-minute working resolution and at the latitudes of southern China, ~30° N) using the spThin 0.2.0 package, retaining only one record per grid cell. After the full cleaning and thinning pipeline, 187 unique occurrence records were retained as the presence input for species distribution modeling.

**Figure 7:**
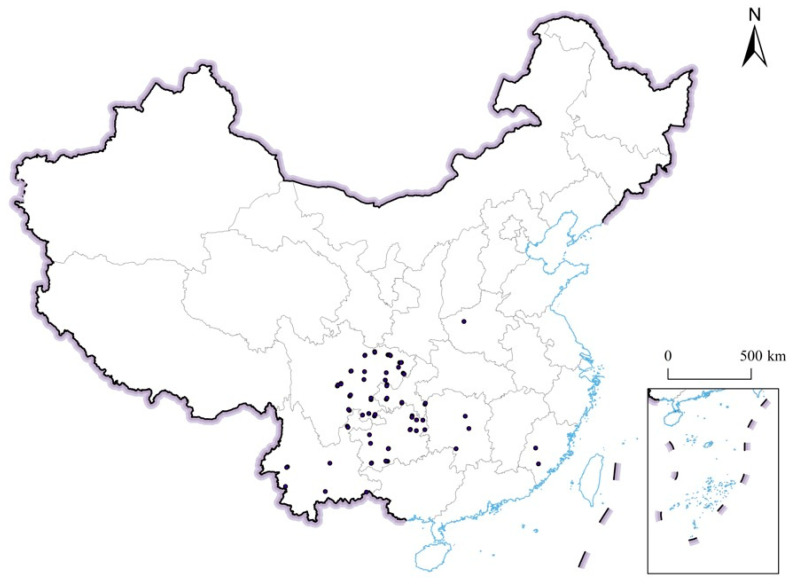
Geographic distribution of the study area and occurrence records of *Bambusa emeiensis* used for species distribution modeling in mainland China. Black dots indicate the final 187 occurrence records retained after taxonomic verification, coordinate cleaning, temporal filtering, and spatial thinning to one record per 2.5-arc-minute grid cell. Grey lines indicate provincial administrative boundaries, and the inset map shows islands and offshore areas included in the national base map. These occurrence records were used as presence data for BIOMOD2 ensemble modeling; pseudo-absence generation, cross-validation, and model evaluation are described in Section 2.3.

The study area was defined as mainland China (approximately 73–135° E, 18–54° N), with all environmental layers clipped to the same extent. All occurrence points were overlaid on the environmental raster stack and on the study-area boundary to confirm that every record fell within a valid, non-NA climate and terrain cell. The final 187 occurrence points were considered to represent the current realized distribution of *B. emeiensis* within the study region and formed the basis for model calibration, cross-validation, and projection under baseline and future climate scenarios (Figure 7).

### 3.2. Environmental Variables and Variable Screening

Environmental predictors were selected to represent the major climatic and topographic constraints on the distribution of *B. emeiensis*. Baseline (1970–2000) climate data were obtained from WorldClim version 2.1 (https://www.worldclim.org) at a spatial resolution of 2.5 arc-minutes (approximately 4.6 km at the study latitudes), comprising all 19 standard bioclimatic variables (bio1–bio19). Elevation (elev) was extracted from the WorldClim v2.1 elevation layer at the same resolution, which was derived from the SRTM 30-arc-second digital elevation model resampled to the working grid. All raster layers were projected to WGS84 geographic coordinates and clipped to the mainland-China extent.

Future climate projections were obtained from the same WorldClim v2.1 archive (CMIP6 downscaled and bias-corrected products) at the identical 2.5-arc-minute resolution. To represent a range of socioeconomic and forcing pathways, three Shared Socioeconomic Pathway (SSP) scenarios were used: SSP1-2.6 (low emissions, sustainability), SSP3-7.0 (medium-to-high emissions, regional rivalry), and SSP5-8.5 (high emissions, fossil-fueled development). Two future time slices were analyzed, corresponding to the 2050s (2041–2060 average) and the 2090s (2081–2100 average), yielding six future scenario–period combinations (SSP126-2050s, SSP126-2090s, SSP370-2050s, SSP370-2090s, SSP585-2050s, and SSP585-2090s). To reduce uncertainty associated with any single general circulation model (GCM), an ensemble of three widely used CMIP6 GCMs was adopted: BCC-CSM2-MR, MIROC6, and CanESM5. For each variable, the multi-model mean across the three GCMs was used as the final future predictor layer while keeping the topographic variable (elev) constant across time periods. The use of only three GCMs was treated as a limitation of the study and is discussed in Section 4.5.

Candidate predictors initially comprised all 19 bioclimatic variables and elevation. To avoid over-parameterization and collinearity-induced instability in parameter estimation, a two-step screening procedure was applied. First, pairwise Pearson correlation coefficients were computed among all candidate predictors using the 187 occurrence cells plus 10,000 random background cells drawn from the study area; for any pair with |*r*| ≥ 0.80, the variable with lower ecological relevance to bamboo growth or with weaker univariate discrimination performance was removed. Second, variance inflation factors (VIFs) were calculated iteratively with the usdm 2.1-6 R package using the vifstep() function, and variables with VIF > 10 were sequentially eliminated. The screening converged on four non-redundant predictors that jointly captured thermal seasonality, topographic heat availability, growing-season moisture supply, and cold-season water conditions: annual temperature range (bio7), elevation (elev), precipitation of the warmest quarter (bio18), and precipitation of the coldest quarter (bio19). These four variables were retained as the final predictor set for all baseline and future projections [15,17,23].

### 3.3. Species Distribution Modeling and Ensemble Construction

Potential habitat suitability of *B. emeiensis* was simulated using an ensemble species distribution modeling framework implemented in the BIOMOD2 R package (version 4.2-5) running under R 4.3.2. Because only presence records were available, pseudo-absences were generated to complement the 187 occurrence points. Three independent pseudo-absence sets were drawn at random from the background of the study area using the ‘random’ strategy, with 10,000 pseudo-absences per set. The ‘random’ strategy was chosen for two reasons. First, given the comparatively narrow realized distribution of *B. emeiensis* within the broad mainland-China study area, random pseudo-absences allowed the models to characterize the broad environmental contrast between the known distributional core and the wider background environment. Second, BIOMOD2 applies equal-prevalence weighting internally: with 187 presences and 10,000 pseudo-absences per set, each presence cell received unit weight while each pseudo-absence received an effective weight of approximately 0.0187 (=187/10,000), so that the summed weights of presences and pseudo-absences were equal within each modeling run [24]. Pseudo-absences were further constrained to cells with valid values for all four predictor layers and outside a 5-km buffer around any presence cell to minimize the chance of sampling false absences within the occupied climatic space.

For each of the three pseudo-absence sets, the occurrence plus pseudo-absence data were partitioned into calibration and evaluation subsets using random split-sample cross-validation: 75% of the data were used for model calibration, and the remaining 25% were used for independent evaluation. This split was repeated five times per pseudo-absence set, yielding 3 × 5 = 15 calibration-evaluation replicates per algorithm. Ten single-model algorithms available in BIOMOD2 were fitted to each replicate: generalized linear models (GLMs), generalized additive models (GAMs), multiple adaptive regression splines (MARS), classification tree analysis (CTA), flexible discriminant analysis (FDA), artificial neural networks (ANNs), surface range envelope (SRE), generalized boosted models (GBMs), random forest (RF), and maximum entropy (MaxEnt). Algorithms were run with BIOMOD2 default parameter settings except where noted: the GLM used a binomial family with logit link, quadratic terms allowed, and stepwise AIC selection; GAM used the mgcv engine with thin-plate regression splines and smoothing parameter selected by GCV; MARS was fitted with a maximum of two-way interactions; CTA used a maximum depth of 25 with 5-fold internal cross-validation for pruning; FDA used MARS as the internal engine; the ANN used a single hidden layer with the number of units selected by 5-fold internal cross-validation; SRE used a quantile of 0.025; the GBM was fitted with 2500 trees, an interaction depth of 7, shrinkage of 0.001, and a bag fraction of 0.5; RF used 500 trees with the default mtry; and MaxEnt was run via the bundled maxent.jar with linear, quadratic, product, and hinge features enabled and a regularization multiplier of 1. Prevalence weighting was fixed at 0.5 in all runs.

Across the 15 replicates, each single-model run produced ROC and TSS evaluation scores on its held-out 25% partition. These scores were then used to construct a weighted-mean ensemble (EMwmean, the BIOMOD_EnsembleModeling ‘EMwmean’ option). Only single-model runs meeting a minimum predictive-performance threshold of TSS ≥ 0.70 were retained for inclusion in the ensemble, and their contributions were weighted proportionally to their TSS scores (decay = ‘proportional’). Algorithms that consistently fell below this threshold were effectively excluded. In practice, this meant that ANN and SRE were down-weighted or dropped in most replicates, while MARS, GAM, CTA, GBM, MaxEnt, RF, FDA, and GLM contributed the bulk of the ensemble signal. The weighted-mean ensemble was then used to predict continuous habitat suitability (scaled to 0–1000 by BIOMOD2 convention) for the baseline and for each of the six future SSP–period scenarios.

### 3.4. Model Evaluation, Variable Importance, and Response Curves

Predictive performance was evaluated using two complementary indices: the area under the receiver operating characteristic curve (ROC/AUC) and the true skill statistic (TSS). For each of the 15 calibration–evaluation replicates, ROC and TSS were computed on the held-out 25% evaluation partition. The mean ± standard deviation of ROC and TSS across the 15 replicates was reported for every single-model algorithm (Table 1). Following common practice in ensemble SDM studies, single-model performance was interpreted as follows: TSS < 0.40 indicated poor, 0.40–0.75 fair-to-good, and >0.75 excellent discrimination [25]; AUC > 0.90 was considered excellent [26]. For the weighted-mean ensemble (EMwmean), the same ROC and TSS were computed, together with the optimal probability threshold maximizing TSS, sensitivity, and specificity (Table 2). Because the occurrence records were spatially structured, these random cross-validation metrics were interpreted as measures of discrimination within the sampled environmental and geographic domain rather than as direct estimates of spatial transferability to novel climates.

Variable importance was quantified within the ensemble model using the BIOMOD2 permutation procedure (BIOMOD_VariablesImportance), in which each predictor was randomly permuted and the correlation between predictions made with the permuted variable and those made with the intact variable was recorded; the importance value was computed as 1—mean correlation, averaged over 10 permutations. Higher values indicate a greater contribution of the variable to the ensemble prediction. Response curves for the four retained predictors were generated with the BIOMOD2 implementation of ‘evaluation strips’ [27], in which each predictor was varied across its full observed range while the remaining predictors were held at their median value over the calibration data, and the resulting ensemble-predicted probability of occurrence was plotted. Because the response of bio19 at the wettest tail was supported by relatively few presence cells, the upper-tail pattern was treated as a potential edge artifact rather than as a robust ecological threshold. The response curves were therefore used primarily for conservative interpretation of the central predictor ranges, while full-range curves are provided for transparency. Together, these procedures were used to evaluate model reliability and to interpret the realized climatic-topographic niche of *B. emeiensis*.

### 3.5. Current and Future Habitat Suitability Projection

The EMwmean ensemble was projected onto the baseline and future environmental layers using BIOMOD_EnsembleForecasting, producing continuous habitat-suitability rasters scaled to 0–1000. The same ensemble structure and weights were applied across all seven scenarios (current, SSP126–2050s, SSP126–2090s, SSP370–2050s, SSP370–2090s, SSP585–2050s, and SSP585–2090s) to ensure that cross-scenario comparisons reflected only changes in climate inputs. The binarization threshold separating suitable from unsuitable habitat was taken as the TSS-maximizing cutoff of the ensemble model (532/1000; see Table 2), following the common practice of using the TSS-optimal threshold for presence-absence conversion [25,28].

To characterize habitat quality structure, the continuous suitability rasters were further classified into four classes using the natural-breaks (Jenks) classification implemented in ArcGIS Pro 3.1, anchored to the TSS threshold so that the unsuitable class terminated at the TSS cutoff. The resulting class boundaries (on the 0–1000 BIOMOD2 scale) were: unsuitable, 0–532; low suitability, 533–650; moderate suitability, 651–800; and high suitability, 801–1000. We use the term ‘moderate suitability’ consistently throughout the manuscript (replacing the inconsistent terminology ‘medium’ and ‘median’ that appeared in earlier drafts). The same class boundaries were applied uniformly to the baseline and to all six future scenarios to allow for a direct comparison across time periods and SSPs. For each scenario, the area (×10^4^ km^2^) and proportion (%) of each suitability class were calculated from the classified rasters using the Albers equal-area conic projection appropriate for China. Future projections were interpreted from two complementary perspectives: (i) changes in total suitable habitat area (sum of low, moderate, and high suitability), and (ii) changes in highly suitable core habitat (the high-suitability class alone), because the latter is more directly related to the persistence of climatically optimal cultivation and conservation zones.

### 3.6. Centroid-Shift Analysis

To quantify the spatial reorganization of habitat under climate change, centroid-shift analysis was performed separately for (i) the overall suitable habitat (low + moderate + high suitability combined), and (ii) the highly suitable core habitat (high-suitability class only). For each scenario, the corresponding binary raster was converted to a polygon feature class, and the geographic centroid was computed as the area-weighted mean of the cell centroids in the Albers equal-area conic projection for China using the Mean Center (Spatial Statistics) tool in ArcGIS Pro 3.1; the resulting centroid coordinates were then back-transformed to WGS84 geographic coordinates for reporting. The SDM Toolbox v2.5 ‘Centroid Changes (Lines)’ tool was used to generate the migration trajectories linking the current centroid to each future centroid. Migration distance (km) between the current centroid and each future centroid was computed as the great-circle (geodesic) distance on the WGS84 ellipsoid, and migration direction was reported as the compass bearing of the displacement vector (measured clockwise from north and classified into eight cardinal and intercardinal sectors: N, NE, E, SE, S, SW, W, NW).

The analysis was conducted separately for overall suitable habitat and highly suitable core habitat because these two components may respond differently to climate change. Overall suitable habitat reflects the broad potential climatic space available to the species, whereas highly suitable core habitat reflects the spatial distribution of the most favorable environments. Comparing centroid shift distance and direction between the two habitat types made it possible to assess whether future change involved simple outward displacement of the suitable range or more complex internal restructuring of habitat quality.

### 3.7. Statistical Analysis and Visualization

All species distribution modeling, ensemble construction, evaluation, variable-importance quantification, and response-curve generation were performed in R 4.3.2 [29] using the biomod2 package (version 4.2-5) with the following supporting packages: raster 3.6-26 and terra 1.7-71 for raster handling, sp 2.1-3 for spatial objects, dismo 1.3-14 for MaxEnt interfacing, gbm 2.1.9, randomForest 4.7-1.1, mgcv 1.9-1, earth 5.3-3, and nnet 7.3-19 for the individual algorithms, usdm 2.1-6 for variance-inflation screening, CoordinateCleaner 2.0-20 for record cleaning, and spThin 0.2.0 for spatial thinning. MaxEnt was run through maxent.jar version 3.4.4. Raster classification into suitability classes, area tabulation in the Albers equal-area conic projection for China, centroid computation, and centroid-shift trajectory generation were performed in ArcGIS Pro 3.1 with the SDM Toolbox v2.5 extension [30]. Habitat-suitability maps, centroid-shift maps, response curves, and bar plots of area statistics were produced in ArcGIS Pro 3.1 and in R using ggplot2 3.4.4. All random-number operations (pseudo-absence sampling and data splitting) were initialized with a fixed seed (set.seed(123)) to ensure reproducibility. Input data, configuration scripts, and archived BIOMOD2 project files are available from the corresponding author upon reasonable request.

## 4. Discussion

### 4.1. Climatic and Topographic Associations with the Distribution of Bambusa emeiensis

Our results indicate that the annual temperature range, elevation, and warm-season precipitation are the strongest statistical correlates of *B. emeiensis* distribution in the ensemble model. Because the present analysis is correlative, these associations should be interpreted as statistical relationships rather than as direct evidence of specific physiological mechanisms. In particular, the dominant role of annual temperature range (bio7) in the variable-importance ranking is partly attributable to its covariation with latitude and continentality across mainland China, and bio7 is well-known to be correlated with several bioclimatic variables that were removed during VIF screening (e.g., mean annual temperature, mean diurnal range). The claim that *B. emeiensis* ‘responds physiologically’ to the amplitude of seasonal thermal fluctuation would require mechanistic data (e.g., controlled-environment thermal-amplitude experiments on culm elongation, photosynthetic safety margins, and rhizome dynamics) that are beyond the scope of a correlative SDM. We therefore restrict our interpretation here to the more conservative statement that the realized distribution of *B. emeiensis* is statistically associated with a climatic gradient correlated with seasonal thermal amplitude.

This pattern is nonetheless ecologically plausible because bamboo growth, culm recruitment, and stand persistence are closely regulated by hydrothermal conditions, while topography modifies local heat balance, moisture retention, and the effective length of the growing season [10,31,32,33]. Elevation likely integrates several limiting processes, including reduced heat accumulation, greater exposure to low-temperature stress, and shorter effective growth periods at higher altitudes, whereas precipitation during the warmest quarter reflects moisture availability during the principal period of bamboo growth and biomass accumulation [1,22,33]. Cold-season precipitation contributed less to the ensemble, and we caution against over-interpreting its upper-tail behavior as an ecological threshold (Section 3.1). Together, these results support the view that the realized distribution of *B. emeiensis* is shaped by the joint action of thermal seasonality, moisture supply, and terrain-mediated environmental heterogeneity rather than by a single climatic factor alone [2,15,17].

### 4.2. Habitat Change as Restructuring Superimposed on Overall Contraction

A central result of this study is that future habitat change in *B. emeiensis* is best described as a combination of overall contraction with strong internal restructuring of habitat quality. Across all six future scenarios examined, the total suitable habitat declined by 25–53% relative to the current climate, while highly suitable core habitat contracted disproportionately (41–95%). Even in scenarios with a comparatively moderate reduction in total area (e.g., SSP370 scenarios, around −25% to −27%), the core-habitat reduction was severe (≥76%). This is the opposite of the pattern that would be expected if climate warming were simply pushing the species into newly available climatic space.

Similar contrasts between overall suitable area and the persistence of optimal habitat have been reported in climate-change assessments of bamboos and other plants, where the contraction of climatic stability or core habitat quality is often more severe than the headline change in total area [2,6,12,13,14,34,35]. For bamboo management, this distinction is especially important because areas classified as broadly suitable are not necessarily equivalent in productivity, regeneration potential, or long-term cultivation value. Studies on bamboo distribution in China likewise show that future climate change may produce nonlinear and scenario-dependent responses, with some species or regions gaining suitable area while others lose their most favorable climatic envelopes or shift toward new environmental space [2,11,14,21,22]. For *B. emeiensis* specifically, our results indicate that the dominant signal across SSPs and time periods is loss of core habitat, and that interpreting future habitat dynamics primarily through changes in total suitable area would substantially underestimate the ecological and management significance of this loss.

### 4.3. Divergent Centroid Shifts Reveal Internal Spatial Reorganization of Optimal Habitat

The asynchronous centroid movements of overall suitable habitat and highly suitable core habitat further support the interpretation that climate change is reorganizing the internal structure of the habitat system rather than merely shifting its external boundary. In species distribution research, centroid displacement is often used as a compact indicator of spatial response to climate change, but centroid trajectories derived from all suitable cells can conceal important differences in the behavior of the most favorable habitat fractions [8,9,23]. In our study, the core-habitat centroid often moved farther, or in a different direction, than the centroid of total suitable habitat, indicating that the geography of optimal environments is changing independently from the broader distribution envelope. Comparable findings have been reported in recent SDM studies showing that future climate change can generate directional divergence, elevational shifts, and the fragmentation of high-quality habitat even where broader suitable areas appear relatively persistent [13,34,35,36].

At the same time, centroid-based summaries must be used with care under scenarios in which the underlying habitat collapses to a small number of cells. The SSP585-2050s core-habitat centroid is the clearest example: because the high-suitability area in this scenario was very small, the corresponding northward displacement should be interpreted as indicative rather than as a precise migration pathway. For *B. emeiensis*, this means that tracking only the outward margin of potential distribution would be insufficient for identifying climatically durable introduction zones or long-term conservation nuclei, but centroid statistics for extreme-contraction scenarios should be treated cautiously.

### 4.4. Implications for Introduction, Expansion Planting, and Germplasm Conservation

A fundamental constraint underlies the management interpretation of all the future-habitat projections presented here. *Bambusa emeiensis* is a sympodial (clumping) bamboo that propagates predominantly by rhizome and clump division and has very limited natural seed dispersal [2,10]. Newly emerging climatically suitable patches that appear hundreds of kilometers from the current centroid—particularly the southwestward and southward shifts of core habitat documented in Section 3.4—are essentially unreachable on management-relevant timescales without human-mediated planting. Projections of ‘potentially suitable habitat’ for this species should therefore be interpreted as a guide to assisted introduction and managed cultivation, and not as predictions of natural range expansion. The following management recommendations are framed within this assisted-migration context.

From this perspective, the results suggest that future planning for *B. emeiensis* should move beyond an area-based view of suitability change and adopt a structure-based strategy centered on core-habitat persistence, connectivity, and managed directional relocation. Stable or relatively persistent highly suitable areas, identified by the overlap of high suitability across multiple future scenarios, should be treated as priority zones for germplasm conservation, ex situ backup planning, and long-rotation cultivation layout, because these areas are most likely to retain the climatic combinations associated with reliable growth and stand performance [9,20,37]. Areas that emerge as moderately suitable under future scenarios may still be valuable, but they are better regarded as candidate zones for phased introduction, provenance testing, and management trials rather than as immediate substitutes for present core habitats [2,21,22]. The strong core-habitat contraction documented here (Section 3.3) reinforces the urgency of identifying and protecting climatically durable core nuclei now, rather than waiting for the projected moderate-suitability areas in newly emerging regions to mature into functional habitat through planting. Conversely, fragmented peripheral habitats should be treated cautiously because apparent suitability gains at the range margin may not translate into long-term establishment success when dispersal limitation, local site conditions, soil constraints, and management feasibility are considered [6,37,38]. This interpretation is consistent with broader calls in climate-adaptation planning to prioritize habitat quality, spatial continuity, and management resilience rather than relying exclusively on gross changes in suitable area [11,14,23].

### 4.5. Methodological Strengths and Limitations

This study benefited from an ensemble SDM framework that combined multiple algorithms and thus reduced dependence on the assumptions and idiosyncrasies of any single model, an approach widely recommended for improving the robustness of distribution forecasts under climate change [18,19,20,39]. The use of multiple evaluation metrics and variable-response analysis also strengthened the ecological interpretability of the projections [25,27,40,41].

Several methodological limitations should nevertheless be made explicit. First, model evaluation was based on repeated random 75/25 cross-validation. Because occurrence records are spatially autocorrelated, random CV may inflate transferability metrics relative to spatially independent validation [42,43]. The ROC and TSS values reported in this study should therefore be interpreted as apparent discrimination performance under the sampled data structure, not as definitive evidence of model transferability under novel climates. Spatial block cross-validation or spatial-buffer validation is a priority for follow-up work.

Second, future projections were based on the multi-model mean of three CMIP6 GCMs. Although this approach reduces dependence on any single climate model, three GCMs remain a relatively small subset of the CMIP6 ensemble. The non-monotonic change in highly suitable core habitat under SSP5-8.5 should therefore be interpreted cautiously, and future work should include a broader GCM ensemble to quantify climate-model uncertainty more fully.

Third, centroid statistics computed over very small high-suitability cell sets are inherently unstable. This limitation is particularly relevant for SSP585-2050s, in which the high-suitability core habitat declined sharply. The corresponding centroid displacement should therefore be interpreted as a qualitative indication of spatial reorganization rather than as a precise migration distance or direction.

Fourth, the ‘random’ pseudo-absence strategy and equal-prevalence weighting were used to represent the broad environmental background across mainland China (Section 2.3). This design is appropriate for broad-scale SDM calibration but cannot fully eliminate the possibility of false absences or sensitivity to pseudo-absence placement. Future work could compare random, disk, SRE, and target-group background strategies when independent information on bamboo-specific environmental constraints becomes available.

Fifth, the bio19 response curve exhibited an abrupt rise at the wettest tail (above ~250 mm) that coincided with sparse sampling of the predictor distribution at presence cells. This is consistent with a possible edge artifact rather than a real ecological threshold, and we therefore interpreted the upper tail of bio19 cautiously. The same caution applies more generally to the upper tails of all four predictors.

Like most correlative SDMs, the present projections assume relative niche conservatism and cannot fully represent adaptive evolution, demographic lag, dispersal limitation, biotic interactions, or management-driven establishment success [16,20,37]. Of these, dispersal limitation is particularly relevant for *B. emeiensis*, as discussed in Section 4.4, and our future-habitat projections should be interpreted under an explicit assisted-migration framing. In addition, although climate and topography captured the major broad-scale constraints on distribution, realized suitability for bamboo can also be modified by soil properties, land use, disturbance regime, and stand-level management, all of which may become increasingly important when model outputs are translated into cultivation decisions [14,21,22]. Future work should therefore integrate finer-resolution edaphic and management variables, broader climate-model ensembles, alternative pseudo-absence strategies, spatially independent validation, and field validation in newly emerging suitable areas to improve the operational value of climate-adaptation recommendations for *B. emeiensis*.

## 5. Conclusions

Using an ensemble species distribution modeling framework, this study showed that the current suitable habitat of *Bambusa emeiensis* is concentrated in southwestern China and is primarily associated with annual temperature range, elevation, and warm-season precipitation, the first of which should be interpreted as a statistical correlate (covarying with latitude/continentality) rather than as a direct physiological driver.

Under all six future SSP–period scenarios examined, the total suitable habitat declined relative to the current climate by 25–53%, and highly suitable core habitat contracted disproportionately by 41–95%; the future of *B. emeiensis* is therefore best described as an overall contraction with strong internal restructuring of habitat quality, rather than as a simple range expansion or contraction.

Centroid shifts of overall suitable habitat and highly suitable core habitat are not always synchronized, indicating that climate change may reorganize not only habitat extent but also the internal geography of optimal environments; centroid statistics for collapsed core-habitat scenarios should be interpreted cautiously because they are sensitive to the spatial configuration of small high-suitability areas.

Because *B. emeiensis* is a clumping bamboo with very limited natural seed dispersal, projections of future ‘suitable habitat’ should be interpreted as guides to assisted introduction and managed cultivation. Management, introduction, and germplasm conservation planning should therefore prioritize the persistence, connectivity, and managed directional relocation of highly suitable core habitats, rather than relying solely on the total suitable area.

## Figures and Tables

**Table 1:** Cross-validation statistics for single models and evaluation metrics for the ensemble model (random 75/25 hold-out cross-validation; mean ± SD across 15 replicates).

Model Algorithm	ROC	TSS
MARS	0.970 ± 0.008	0.902 ± 0.023
GAM	0.963 ± 0.009	0.888 ± 0.019
CTA	0.950 ± 0.024	0.883 ± 0.034
GBM	0.969 ± 0.007	0.878 ± 0.046
MaxEnt	0.975 ± 0.006	0.875 ± 0.035
RF	0.988 ± 0.007	0.871 ± 0.068
FDA	0.960 ± 0.018	0.858 ± 0.050
GLM	0.961 ± 0.006	0.818 ± 0.032
ANN	0.783 ± 0.056	0.564 ± 0.108
SRE	0.757 ± 0.025	0.514 ± 0.049

**Table 2:** Evaluation metrics of the weighted-mean ensemble model (EMwmean) for *Bambusa emeiensis* (random hold-out cross-validation).

Evaluation Metric	Threshold	Sensitivity (%)	Specificity (%)	Score
TSS	532.000	97.531	93.900	0.914
AUCroc	581.000	96.914	94.300	0.989

**Table 3:** Importance ranking of key environmental variables in the ensemble model.

Rank	Environmental Variable	Full Variable Name	Mean Variable Importance
1	bio7	Annual temperature range	0.403
2	elev	Elevation	0.288
3	bio18	Precipitation of warmest quarter	0.190
4	bio19	Precipitation of coldest quarter	0.049

**Table 4:** Area statistics of habitat suitability classes of *Bambusa emeiensis* under the current climate.

Suitability Class	Area (×10^4^ km^2^)	Proportion (%)
Unsuitable	829.46	87.37
Low suitability	31.35	3.30
Moderate suitability	52.23	5.50
High suitability	36.31	3.82
Total suitable habitat	119.89	12.63

**Table 5:** Changes in habitat suitability area of *Bambusa emeiensis* under current and future climate scenarios. Rate of change was computed relative to the current climate row.

Climate Scenario	Low Suitability Area (×10^4^ km^2^)	Moderate Suitability Area (×10^4^ km^2^)	High Suitability Area (×10^4^ km^2^)	Total Suitable Area (×10^4^ km^2^)	Rate of Change from Current
Current (2020)	31.35	52.23	36.31	119.89	—
SSP126–2050s	29.93	32.99	21.28	84.20	−29.77%
SSP126–2090s	23.88	35.31	11.72	70.91	−40.85%
SSP370–2050s	33.97	46.26	7.70	87.93	−26.66%
SSP370–2090s	36.93	43.68	8.75	89.36	−25.47%
SSP585–2050s	24.17	31.03	1.67	56.87	−52.56%
SSP585–2090s	29.61	50.99	5.89	86.49	−27.86%

**Table 6:** Centroid shift distance and direction for overall suitable habitat and highly suitable core habitat.

Climate Scenario	Overall Shift Distance (km)	Overall Direction	Core Shift Distance (km)	Core Direction
Current (2020)	0.00	Original	0.00	Original
SSP126–2050s	92.51	Southwest	178.69	Southwest
SSP126–2090s	91.01	East	123.58	Southwest
SSP370–2050s	96.55	East	160.99	Southwest
SSP370–2090s	97.70	Southwest	324.63	Southwest
SSP585–2050s	163.45	East	44.64	North *
SSP585–2090s	188.01	Southeast	313.59	South

* The SSP585-2050s core-habitat centroid was computed from only 1.67 × 10^4^ km^2^ of highly suitable cells, so the reported displacement should be interpreted as indicative rather than precise.

## Data Availability

The complete dataset is available from the corresponding author upon reasonable request and will be deposited in a public repository upon acceptance.
